# Herpes zoster–induced critical rhabdomyolysis with secondary hypothyroidism: a case report

**DOI:** 10.3389/fmed.2026.1969501

**Published:** 2026-10-27

**Authors:** Xiangkang Lv, Bin Zhang, Wei Yang, Xiao Lu

**Affiliations:** Department of Emergency Medicine, Second Affiliated Hospital, Zhejiang University School of Medicine, Hangzhou, China

**Keywords:** acute kidney injury, herpes zoster, hypothyroidism, multidisciplinary care, rhabdomyolysis, varicella-zoster virus

## Abstract

Primary varicella-zoster virus (VZV) reactivation causes herpes zoster (HZ), typically presenting with dermatomal rash and neuropathic pain. Rhabdomyolysis as a systemic complication of HZ is extremely rare, and its underlying pathogenesis and optimal management remain poorly characterized, especially in elderly patients. Here we report a 63-year-old female who presented with fever, generalized myalgia, and oliguria, with serum creatine kinase (CK) rapidly escalating from 4,383 U/L to a peak of 186,383 U/L. Further investigation revealed a preceding history of lumbar herpes zoster two weeks prior, accompanied by virus-induced secondary hypothyroidism. Muscle biopsy confirmed extensive myofiber necrosis without inflammatory infiltration, consistent with non-inflammatory rhabdomyolysis. The patient developed acute kidney injury, hepatic dysfunction, and respiratory failure, and was managed with multidisciplinary intensive care including mechanical ventilation, 9 days of continuous renal replacement therapy, glucocorticoid anti-inflammatory therapy, and levothyroxine replacement. After 12 days of treatment, CK decreased to 3,381 U/L with significant recovery of multiorgan function, and the patient was transferred out of the intensive care unit. This case highlights that HZ can trigger life-threatening rhabdomyolysis, possibly via VZV-mediated immune dysregulation and secondary hypothyroidism. Detailed history taking, early CK screening, and multidisciplinary organ support are critical to improve outcomes in this rare but severe condition.

## Introduction

Primary varicella-zoster virus (VZV) infection typically causes chickenpox in children, with severe complications being uncommon. In contrast, VZV reactivation in adults, especially the elderly and immunocompromised, manifests as herpes zoster (HZ) and carries a higher risk of systemic involvement and serious sequelae (1). The classic features of HZ are dermatomal vesicles and neuralgic pain, and clinical attention is often focused on postherpetic neuralgia and neurological complications.

Rhabdomyolysis secondary to VZV infection, however, is an extremely rare critical complication (2). While non-traumatic rhabdomyolysis is most frequently triggered by influenza or coxsackievirus, VZV-associated cases are scarcely reported, and the underlying mechanisms, clinical characteristics, and risk factors in the elderly population remain poorly elucidated (3). This complication can progress rapidly, leading to acute kidney injury (AKI), severe electrolyte disturbances, and even multiorgan dysfunction. In older patients, blunted symptoms and resolved skin lesions often result in missed or delayed diagnosis.

Herein, we describe a 63-year-old patient with HZ complicated by severe rhabdomyolysis and secondary hypothyroidism, and review the relevant literature to enhance clinicians' awareness of this rare but devastating entity and to provide practical guidance for early recognition and multidisciplinary management.

## Case presentation

### Patient information and clinical history

A 63-year-old female was admitted to the emergency intensive care unit (EICU), with a 6-day history of fever and generalized myalgia, and a 4-day history of progressive oliguria. She explicitly denied any prior history of thyroid disease or use of thyroid-related medications. She denied exposure to high-temperature environments within the preceding 2 weeks. She had no history of trauma, strenuous exercise, toxic exposure, or familial myopathy.

Two weeks prior to admission, the patient developed a localized painful erythematous rash over the left T12 dermatome (thoracic 12 level, lumbar region), which was clinically diagnosed as herpes zoster at a local clinic. She was treated with topical acyclovir plus oral adjuvant symptomatic medications for herpes zoster, with pain relief within 3–4 days and residual cutaneous hyperpigmentation; no new lesions appeared. Six days before presentation, she developed fever with cough, sputum production, and diffuse muscle soreness. She received empirical azithromycin and symptomatic treatment at a local hospital, but her symptoms did not improve. Over the following four days, fever subsided but myalgia worsened markedly, accompanied by generalized edema and a sharp decline in urine output, with urine turning wine-colored. Serial testing showed serum creatine kinase (CK) rising from 4,383 U/L to 32,183 U/L within 48 h. She was then transferred to our hospital for further management.

### Physical examination on admission

On admission, vital signs were: temperature 37.0 °C, pulse 91 bpm, respiratory rate 17 breaths/min, blood pressure 145/73 mmHg, and oxygen saturation 96% on nasal cannula oxygen. The patient was conscious but lethargic. Physical examination revealed severe facial and extremity edema, proximal muscle weakness (grade 3/5 in all four limbs), and coarse breath sounds in both lungs. Residual brownish hyperpigmentation was noted over the left T12 dermatome, consistent with healed herpes zoster. No active skin vesicles or focal neurological deficits were observed.

### Diagnostic investigations

#### Laboratory findings

1. Muscle injury markers: Serum CK rose progressively from 4,383 U/L (2 days pre-admission) to a peak of 186,383 U/L on the day of admission; CK-MB 1,079 ng/mL; lactate dehydrogenase 3,807 U/L; aspartate aminotransferase (AST) 2,070 U/L; alanine aminotransferase (ALT) 565 U/L; troponin-I 0.374 ng/mL.
2. Urinalysis: Occult blood 3+, protein 2+, and markedly elevated erythrocytes, consistent with myoglobinuria. Urine crystal count <1 per microliter.
3. Renal function and serum electrolytes: Serum creatinine 67.8 μmol/L, blood urea nitrogen (BUN) 8.82 mmol/L, sodium 121.9 mmol/L, potassium 4.19 mmol/L, calcium 1.96 mmol/L. Fractional excretion of sodium (FeNa), calculated from paired serum and urine sodium-creatinine measurements on admission, was 0.64%.
4. Inflammatory markers: C-reactive protein (CRP) 147.4 mg/L, procalcitonin (PCT) 2.98 ng/mL, interleukin-6 (IL-6) 398 pg/mL.
5. Thyroid function tests: TSH 6.24 mIU/L (reference: 0.60–4.40 mIU/L), FT3 1.67 pmol/L (reference: 3.45–5.50 pmol/L), FT4 8.17 pmol/L (reference: 11.00–17.70 pmol/L). Anti-thyroglobulin antibody (ATGAb) and thyroid peroxidase antibody (TPOAb) were within normal limits.
6. Autoimmune myositis panel: All 44 myositis-specific and myositis-associated antibodies were negative.
7. Coagulation: Elevated fibrinogen and D-dimer, indicating a hypercoagulable state.

#### Imaging and pathological examinations

1. Chest CT: Patchy inflammatory exudation in the right lung, dependent atelectasis, and small pericardial effusion.
2. Abdominal contrast-enhanced CT: Mild perinephric and peripancreatic exudation. The liver, adrenal glands, and other visualized abdominal organs demonstrated no remarkable abnormalities.
3. Thigh MRI: Subcutaneous edema and intramuscular fluid collection, without focal inflammatory masses (Figure 1).
4. Muscle biopsy (right biceps brachii, HE stain): Extensive myofiber necrosis, degeneration and lysis; marked variation in fiber size with scattered atrophic fibers; prominent endomysial edema; no inflammatory cell infiltration (Figures 2A,B). These findings confirmed rhabdomyolysis and excluded autoimmune necrotizing myositis.

**Figure 1:**
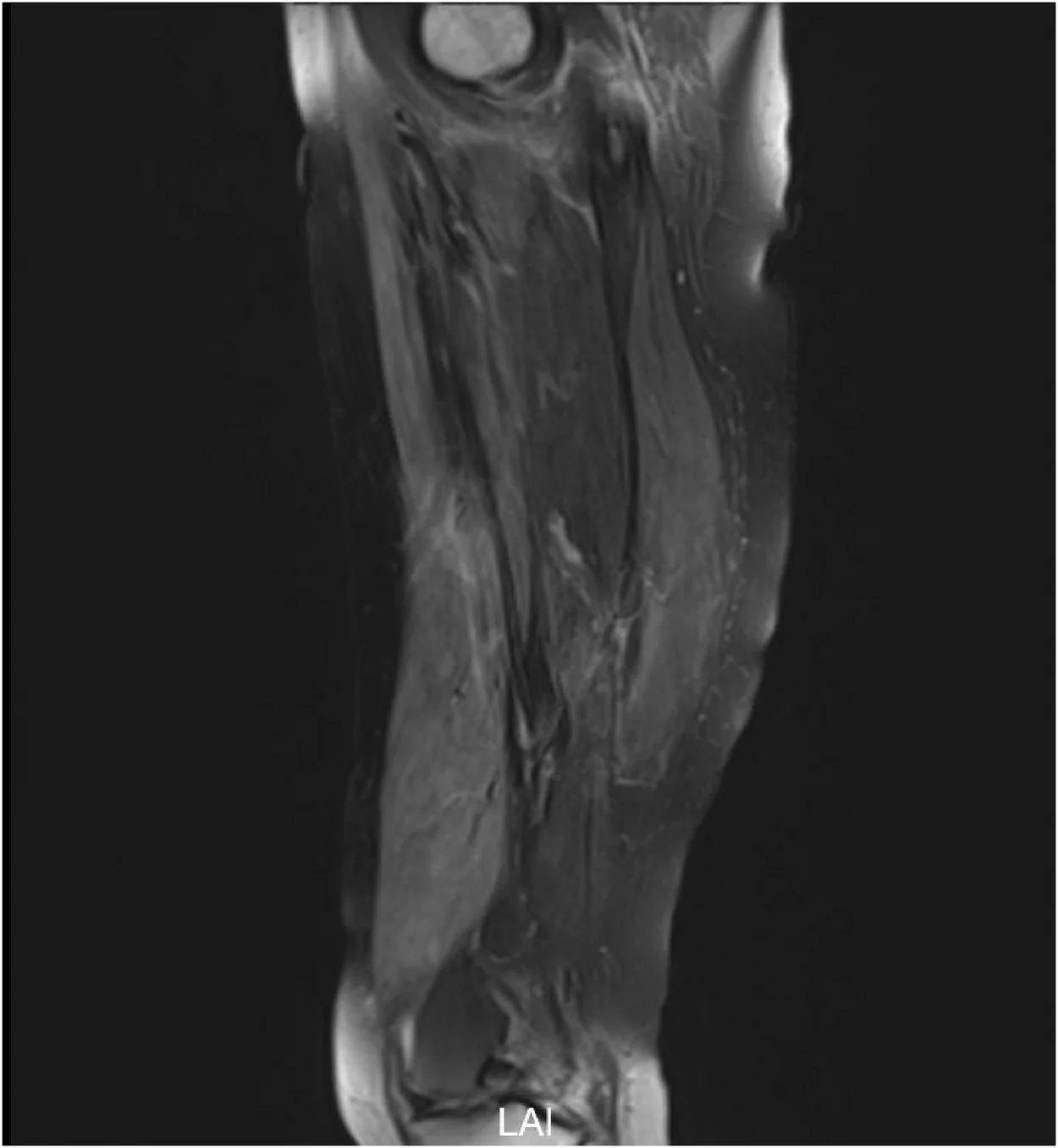
Thigh MRI showing diffuse muscle edema and suspected necrotic changes. Coronal T2-weighted image of the left thigh demonstrates extensive subcutaneous edema, intramuscular fluid collection, and swollen muscles with heterogeneous high signal intensity in the quadriceps and posterior thigh muscles, indicating widespread inflammatory edema and possible necrotic myopathy.

**Figure 2:**
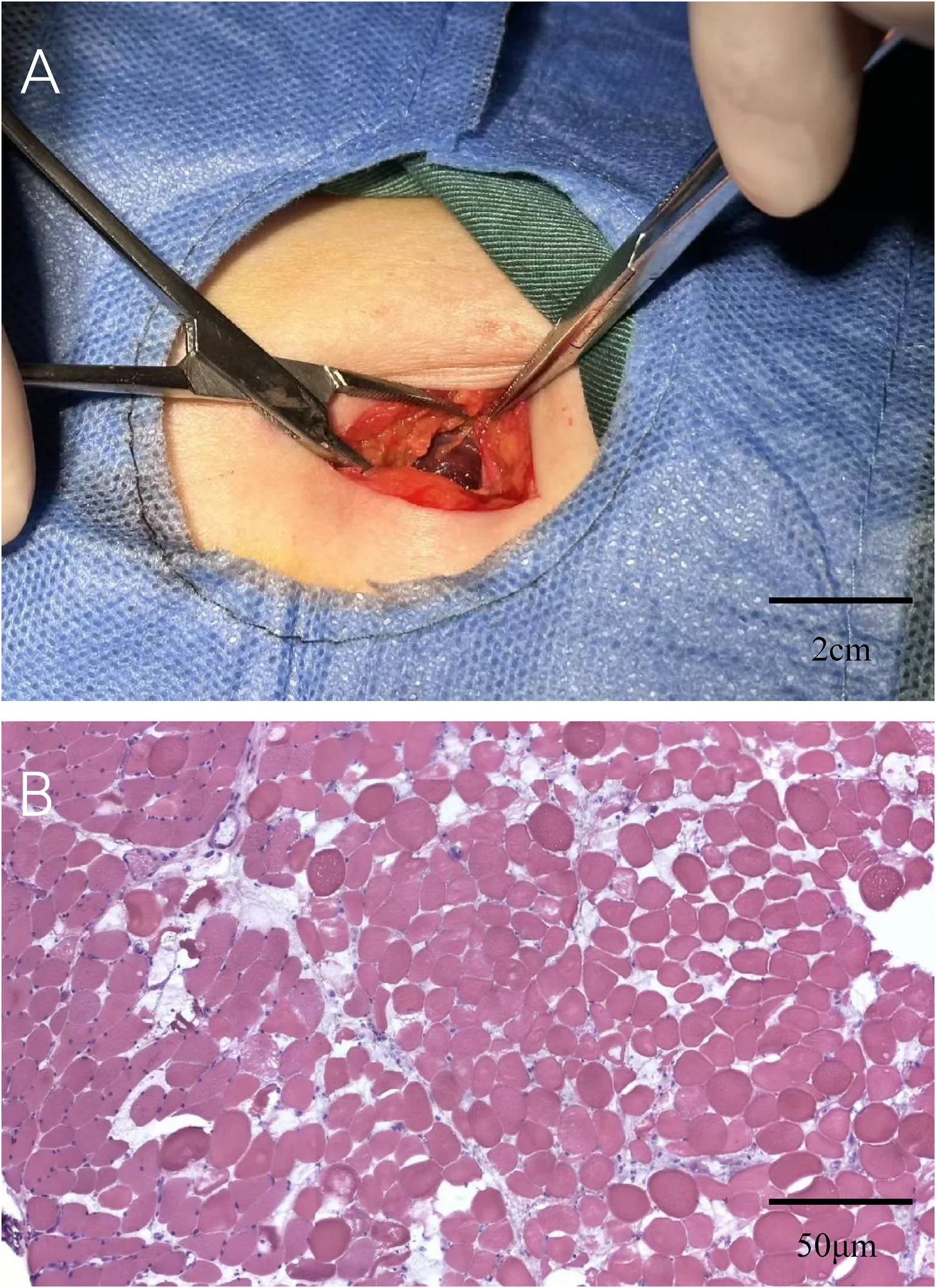
Gross and histopathological findings of the right biceps brachii biopsy. **(A)** Intraoperative gross view of the biceps brachii muscle. The muscle tissue shows dark-red discoloration and diffuse edema, with loss of normal muscle gloss, which is macroscopically consistent with myonecrosis and interstitial edema secondary to rhabdomyolysis. Scale bar, 2 cm. **(B)** Histopathological image under hematoxylin-eosin staining (original magnification ×200). Extensive myofiber necrosis, degeneration and lysis are visible, with marked variation in fiber size and scattered small round atrophic fibers. Endomysial edema is prominent, and no significant inflammatory cell infiltration is observed. Scale bar, 50 μm.

#### Final diagnoses

1. Herpes zoster–induced critical rhabdomyolysis
2. VZV-triggered secondary hypothyroidism
3. Acute kidney injury (stage 3)
4. Acute hepatic and myocardial injury
5. Aspiration pneumonia
6. Multiple electrolyte disturbances and hypoalbuminemia
7. Essential hypertension

#### Therapeutic intervention and clinical course

The patient was immediately classified as critically ill, and a multidisciplinary team (MDT) including intensive care, nephrology, infectious diseases, rheumatology, endocrinology, and rare disease specialists was convened.

1. Respiratory support: Due to progressive hypoxemia and decreased consciousness, emergency endotracheal intubation and mechanical ventilation were performed on admission day. On hospital day 6, percutaneous tracheostomy was performed under bronchoscopic guidance for anticipated prolonged ventilatory support.
2. Renal replacement therapy: A femoral venous dialysis catheter was placed, and continuous veno-venous hemodiafiltration (CVVHDF) was initiated for 9 consecutive days to clear myoglobin, correct electrolyte disturbances, and maintain fluid balance.
3. Anti-infective therapy: Empirical piperacillin-tazobactam was initiated, then escalated to meropenem 1 g every 8 h for persistent pulmonary infection.
4. Etiological targeted therapy: Low-dose methylprednisolone (40 mg/day) was administered for systemic immune dysregulation, and levothyroxine replacement was started to correct hypothyroidism and improve skeletal muscle mitochondrial metabolism.
5. Supportive care: Early enteral nutrition via nasointestinal tube, and physical thromboprophylaxis were implemented.

### Outcome and follow-up

After 12 days of comprehensive EICU management, the patient's clinical condition improved substantially. Generalized edema resolved, and muscle strength recovered. Serial serum CK, lactate dehydrogenase (LDH), and AST were dynamically monitored during hospitalization. CK peaked at 186,383 U/L on admission, accompanied by marked elevation of LDH (5,637 U/L) and AST (2,854 U/L). All three muscle injury markers gradually declined following initiation of CRRT. By hospital day 12, CK decreased to 3,381 U/L, LDH to 414 U/L, and AST to 89 U/L. Of note, baseline serum creatinine at admission was within normal limits (67.8 μmol/L), confirming that severe rhabdomyolysis preceded the subsequent acute kidney injury. During CRRT support, serum creatinine and BUN remained relatively stable. After CRRT cessation on hospital day 10, a prominent rebound in creatinine and BUN was observed, with creatinine reaching a maximum of 359.6 μmol/L on hospital day 12, followed by partial recovery on the same day. Urine output remained severely reduced in the early hospital course and gradually improved from hospital day 8 onward (Table 1). The patient was successfully weaned from mechanical ventilation and transferred to a general ward. No recurrence was observed during follow-up.

**Table 1:** Serial changes of muscle injury markers, renal function parameters, and urine output during hospitalization.

Hospital day	CK (U/L)	LDH (U/L)	AST (U/L)	Creatinine (μmol/L)	BUN (mmol/L)	Urine output (mL/24 h)	CRRT status
HD 1	186,383	5,637	2,854	67.8	8.82	8	CRRT initiated
HD 2	151,923	3,823	2,789	99.8	8.19	3	CRRT (day 2)
HD 3	106,128	3,124	3,195	104.6	9.17	0	CRRT (day 3)
HD 4	96,801	2,154	1,734	104.1	9.07	34	CRRT (day 4)
HD 5	55,622	1,630	1,003	116.0	10.87	21	CRRT (day 5)
HD 6	28,807	1,297	583	108.5	13.59	31	CRRT (day 6)
HD 7	13,964	1,051	367	105.1	13.39	80	CRRT (day 7)
HD 8	7,122	962	233	101.8	13.14	470	CRRT (day 8)
HD 9	3,808	789	160	103.8	14.24	345	CRRT (day 9)
HD 10	2,499	650	111	105.5	11.68	356	CRRT discontinued (ultrafiltration = 0)
HD 11	1,774	503	118	233.3	25.5	141	Post-CRRT; creatinine rebound
HD 12	3,381	414	89	359.6	41.96	N/A	Peak creatinine

CK, creatine kinase; LDH, lactate dehydrogenase; AST, aspartate aminotransferase; BUN, blood urea nitrogen; CRRT, continuous renal replacement therapy; HD, hospital day; N/A, not available.

Reference ranges: CK 40–200 U/L; LDH < 250 U/L; AST < 40 U/L; Creatinine 41–81 μmol/L; BUN 3.1–8.8 mmol/L.

## Discussion

Herpes zoster is a common disease caused by VZV reactivation, predominantly affecting elderly and immunocompromised individuals. While postherpetic neuralgia and cutaneous superinfection are well-known complications, fulminant rhabdomyolysis represents an extremely rare, life-threatening systemic sequela (4). Most VZV-associated rhabdomyolysis cases occur during primary varicella infection in children; severe rhabdomyolysis triggered by zoster reactivation is scarcely reported.

Multiple interconnected mechanistic pathways may explain the extensive muscle injury observed in this patient.

VZV is neurotropic and spreads along sensory nerves to produce skin lesions after reactivation. In our biopsy, we observed widespread myofiber necrosis without prominent inflammatory infiltrates, and we lacked direct PCR or immunohistochemical proof of replicating VZV within muscle tissue. This histology differs from typical influenza-associated viral myitis, which often shows intramuscular inflammatory infiltration and detectable viral antigens inside damaged myofibers. Therefore, direct VZV lytic infection of muscle fibers is unlikely to be the primary driver of injury in this case (5).

VZV reactivation can trigger robust systemic immune activation and release of pro-inflammatory mediators such as tumor necrosis factor-α (TNF-α) and IL-6 (5). In keeping with this mechanism, our patient demonstrated markedly elevated admission inflammatory biomarkers, including interleukin-6 (398 pg/mL), CRP (147.4 mg/L) and PCT (2.98 ng/mL). Circulating cytokines may impair sarcolemmal integrity, disturb intracellular calcium homeostasis and induce myofiber necrosis without marked local muscle inflammation (6). This cytokine-driven pattern matches our biopsy feature of extensive necrosis with sparse inflammatory cell infiltration, representing non-inflammatory rhabdomyolysis.

Hypothyroidism impairs skeletal-muscle mitochondrial metabolism, reduces ATP turnover, alters intramuscular glycogen storage and destabilizes muscle cell membranes (7). Isolated hypothyroidism rarely causes fulminant rhabdomyolysis with extremely elevated CK. In this patient, secondary thyroid dysfunction likely acted as a permissive co-factor, lowering the threshold for muscle damage and rendering myofibers more susceptible to inflammatory stress from VZV-related immune activation.

We propose a synergistic injury model for this case. The index event was T12-dermatome VZV reactivation. Systemic pro-inflammatory cytokine release constituted the primary muscle insult. Concurrent inflammation-driven secondary hypothyroidism further aggravated muscle vulnerability. Since only topical acyclovir plus oral symptomatic agents were administered and urinary crystal studies excluded acyclovir crystal nephropathy, drug-mediated muscle or renal injury was ruled out (8). Acute kidney injury developed secondary to massive rhabdomyolysis-related myoglobin toxicity rather than acting as an initiating trigger (7). The combination of cytokine-mediated stress plus hypothyroid metabolic decompensation together produced the extreme CK elevation. Diagnostically, this case delivers important clinical warnings.

Herpes-zoster-related rhabdomyolysis may present after skin rash has resolved, and can be easily misdiagnosed when complicated by concurrent pneumonia and AKI (9). Diagnosis relies on careful history-taking, recognition of residual dermatomal hyperpigmentation, muscle biopsy, exclusion of autoimmune myositis, and thyroid-function assessment (10). Elderly HZ patients with persistent generalized myalgia, fatigue or tea-colored urine should receive prompt CK and thyroid-function testing.

Clinicians should recognize that severe systemic zoster complications may arise even after cutaneous lesions resolve (11). Persistent generalized myalgia in HZ patients warrants immediate CK testing regardless of blister status. Markedly elevated CK should prompt thyroid-function evaluation to identify potentiating secondary thyroid dysfunction. Once severe rhabdomyolysis develops, multidisciplinary organ support including timely CRRT is critical. Distinguishing myoglobin-induced AKI from drug-crystal nephropathy using FeNa and urinary crystal assessment is essential in critically-ill patients.

This single-case report cannot establish definitive causal relationships between VZV reactivation, secondary hypothyroidism and fulminant rhabdomyolysis, only mechanistic plausibility. We did not perform VZV PCR or viral immunohistochemistry on muscle specimens to fully exclude low-level intramuscular viral presence. Although admission-level pro-inflammatory markers (IL-6, CRP, PCT) confirmed severe systemic inflammation, serial longitudinal cytokine measurements during hospitalization were unavailable to track temporal correlation with muscle injury progression. Furthermore, we cannot completely separate secondary hypothyroid changes from features of non-thyroidal illness syndrome induced by critical systemic inflammation.

Further work is needed to characterize zoster-associated rhabdomyolysis. Prospective studies could evaluate the clinical utility of routine CK screening among elderly HZ patients with prominent myalgia for early detection of subclinical muscle damage. Translational and case-series research incorporating muscle viral testing and serial cytokine profiling will help validate this synergistic injury model and clarify immune links between VZV reactivation, transient thyroid dysfunction and skeletal-muscle necrosis. Such data may inform future risk-stratification and therapeutic strategies.

Therapeutically, this case highlights the value of multidisciplinary team care for rare critical disorders. Beyond standard rhabdomyolysis management (fluid resuscitation, urinary alkalinization, CRRT), low-dose glucocorticoids may suppress excessive systemic inflammation, while levothyroxine replacement can reverse muscle metabolic impairment and accelerate biochemical recovery (12).

## Conclusion

Herpes zoster-induced rhabdomyolysis is a rare but severe complication with rapid progression and high risk of multi-organ failure. Pathogenesis likely involves synergistic effects of VZV-triggered immune dysregulation and secondary hypothyroidism. Clinicians should maintain high vigilance in elderly HZ patients. Detailed history-taking, early biomarker screening and multidisciplinary integrated treatment are keys to early diagnosis and improved prognosis.

## Data Availability

The original contributions presented in the study are included in the article/Supplementary Material, further inquiries can be directed to the corresponding author.
